# Self-weighing among young adults: who weighs themselves and for whom does weighing affect mood? A cross-sectional study of a population-based sample

**DOI:** 10.1186/s40337-021-00391-y

**Published:** 2021-03-10

**Authors:** Samantha L. Hahn, Carly R. Pacanowski, Katie A. Loth, Jonathan Miller, Marla E. Eisenberg, Dianne Neumark-Sztainer

**Affiliations:** 1 Division of Epidemiology & Community Health, School of Public Health, University of Minnesota, Minneapolis, MN USA; 2 Department of Psychiatry & Behavioral Sciences, Medical School, University of Minnesota, Minneapolis, MN USA; 3 Department of Behavioral Health and Nutrition, University of Delaware, Newark, DE USA; 4 Department of Family Medicine and Community Health, Medical School, University of Minnesota, Minneapolis, MN USA; 5 Department of Pediatrics, Medical School, University of Minnesota, Minneapolis, MN USA

**Keywords:** Self-weighing, Self-monitoring, Mood, Young adults, Weight management, Eating disorders

## Abstract

**Background:**

Self-weighing is widespread among young adults and is sometimes recommended by healthcare providers for weight management. The present study aims to deepen our understanding of who is frequently self-weighing among young adults, and to examine for whom self-weighing impacts mood based on weighing frequency and other eating and weight-related characteristics.

**Methods:**

Survey data were collected from a large population-based sample of young adults (31.1 ± 1.6y) participating in Project EAT-IV (*n* = 1719). Cross-sectional data were stratified across sex and analyzed with chi-square, t-tests, and linear and logistic regressions controlling for age, ethnicity/race, education level, and income.

**Results:**

Self-weighing frequency was higher among male and female young adults with a current eating disorder, those trying to lose weight or who endorsed any disordered eating behaviors or cognition, and females with higher BMI. Young adult females were significantly more likely than males to report that self-weighing impacted their mood (53% vs 27%, *p* < 0.05). Among both male and female young adults, there was a higher probability of participants reporting that self-weighing impacted their mood among those who were self-weighing more frequently, had higher BMI, were trying to lose weight, and endorsed disordered eating behaviors or cognitions.

**Conclusion:**

Findings suggest that for many young adults, particularly females and those with weight-related concerns, self-weighing is a behavior that comes with emotional valence. The emotional consequences of self-weighing should be considered when making public health and clinical recommendations regarding the usefulness of self-weighing.

## Plain English summary

Many young adults regularly weigh themselves, sometimes due to the recommendation of a healthcare professional. However, little is known about who weighs themselves and how weighing oneself impacts one’s mood. This study examines who tends to weigh themselves and for whom weighing themselves impacts their mood. Results from 1719 young adults from the general population showed that 53% of young women and 27% of young men reported that weighing themselves impacted their mood. Those with a current eating disorder, who had eating disorder symptoms, or had a higher BMI weighed themselves more frequently. Of concern, young adults who weighed themselves more frequently, had a higher BMI, or had eating disorder symptoms were also more likely to report that weighing themselves impacted their mood. These results suggest that for many young adults, weighing themselves has emotional consequences which could have further downstream health consequences such as engagement in disordered eating.

## Background

Frequent self-weighing is often recommended in the context of weight management under the premise that self-weighing will increase awareness of how one’s behaviors affect weight, leading to behavior change, and ultimately to weight maintenance or loss [1–3]. However, there is a growing body of evidence suggesting that self-weighing may not always be helpful for weight maintenance and may actually be associated with harmful consequences. For example, longitudinal studies have found that adolescents and young adults who self-weighed more frequently at baseline experienced more weight gain over time [4, 5]. Further, longitudinal and experimental studies among adolescents and young adults have also found frequent self-weighing to be associated with decreased self-esteem, and increased anxiety and depression [6, 7]. Cross-sectional and longitudinal studies have also found that more frequent self-weighing is associated with increased likelihood of using unhealthy weight control behaviors [4, 5, 7–9]. Unhealthy weight control behaviors are of particular public health and clinical concern given associations between unhealthy weight control behaviors and numerous negative health consequences including the development of clinically significant eating disorders [10, 11], increased weight gain over time [12], and substance abuse [13, 14]. Because frequent self-weighing is a risk factor for unhealthy weight control behaviors, understanding who is engaging in frequent self-weighing is critical to developing relevant prevention strategies.

One mechanism by which self-weighing is thought to lead to unhealthy weight control behaviors is through self-weighing’s effect on mood. While self-weighing may be useful as a tool in weight regulation if it is received by the person as information, it may be harmful if the information leads to changes in mood, whether positively or negatively. Namely, mood changes due to self-weighing likely serve as a mediator between self-weighing and unhealthy weight control behaviors [7], as it is well established that both positive and negative or variable psychological states, including mood, are precursors to unhealthy weight control behaviors [11, 15–17]. Therefore, to understand for whom self-weighing may be most harmful, it is important to understand for whom self-weighing affects mood. Individuals more likely to be concerned with shape and weight (e.g. females, those of higher body mass index (BMI), those having experienced an eating disorder) may be at heightened risk for self-weighing impacting their mood, though little work has been done to examine these relationships [18, 19]. Understanding for whom self-weighing elicits an emotional response is an important first step in disentangling the complex cyclical relationships between thoughts, mood, behaviors, and weight [20].

It is also likely that the relationships between self-weighing and mood differ between males and females due to differences in societal pressure regarding weight. Among females, there is greater societal pressure to be thin and an increased likelihood of internalizing the thin ideal [21, 22], while among males there is a desire for leanness and simultaneous desire for increased muscularity [23]. The pressure for females to be lower weight specifically may result in females being less likely to view self-weighing objectively. Ultimately, less objectivity about weight may lead to females being more likely to have changes in mood due to self-weighing. Mintz et al. found that in a sample of female college students, approximately 70% weighed themselves and more than half reported that seeing the number on the scale impacted their mood [24]. However, because this sample was exclusively female college students, and was predominantly white, results may not hold true in the general population. Understanding who may be self-weighing and the factors that make an individual more likely to have their mood impacted by the practice of self-weighing will help inform future research to elucidate these relationships, as well as future public health and clinical recommendations regarding self-weighing.

The current study therefore aims to: 1) identify correlates of weighing frequency in a large population-based sample of young adult males and females; 2) determine whether self-weighing frequency is associated with reporting mood changes due to self-weighing among young adult males and females; and 3) examine whether certain eating and weight-related characteristics (lifetime eating disorder diagnosis history, weight management goals, engagement in disordered eating behaviors and cognitions, and BMI percentile) are associated with differing likelihood of mood changes due to self-weighing among young adult males and females.

## Methods

### Sample characteristics

Data for this cross-sectional analysis were collected as part of Project EAT (Eating and Activity in Teens and Young Adults)-IV, the fourth wave of a population-based study designed to examine dietary intake, physical activity, weight management behaviors, weight status, and associated factors in young adults. At the original assessment (1998–1999), a total of 4746 middle and senior high school students at 31 public schools in the Minneapolis-St. Paul metropolitan area of Minnesota completed surveys and anthropometric measures [25, 26]. Fifteen years later (2015–2016), original participants who had responded to at least one previous follow-up survey and could be contacted by mail (*n* = 2770) were mailed letters inviting them to complete the Project EAT-IV survey and a food frequency questionnaire. The selection of survey items for Project EAT-IV was guided by previous Project EAT surveys, formative focus groups with young adults, social cognitive theory and a life course perspective [27]. Scale psychometric properties were examined in the full EAT-IV survey sample and estimates of item test-retest reliability, reported below, were determined in a subgroup of 103 participants who completed the EAT-IV survey twice within a period of 1 to 4 weeks.

Complete EAT-IV survey data were collected online, by mail or by phone from 66.1% of those for whom correct contact information was available (*n* = 1830). Participants were excluded from the present analyses if they had missing data necessary to answer the stated research question for the present study. Specifically, participants were excluded from the current analysis if they were pregnant (*n* = 84), missing BMI measurement (*n* = 20) or missing responses to self-weighing measures including frequency of self-weighing or of self-weighing’s impact on mood (*n* = 7) for a final analytic sample of 1719 with a mean age of 31.0 years (SD = 1.6, range: 25–36). Individuals who were pregnant were excluded because we believed the relationships may differ based on pregnancy status [28]. Because attrition from the original school-based sample did not occur at random, in all analyses, the data were weighted using the response propensity method [29]. Response propensities (i.e., the probability of responding to the EAT-IV survey) were estimated using a logistic regression of response at 15-year follow-up on many predictor variables from the EAT-I baseline survey. The weighting method resulted in estimates representative of the demographic make-up of the original school-based sample, thereby allowing results to be more fully generalizable to the population who were middle- and high school students in the Minneapolis-St. Paul metropolitan area in 1998–1999. All study protocols were approved by the University of Minnesota’s Institutional Review Board Human Subjects Committee.

### Self-weighing measures

Frequency of Self-Weighing was measured with the question: “How often do you weigh yourself?” (test-retest correlation: *r* = 0.91). This item had seven response options ranging from “less than once a month” to “more than once a day”. This question was adapted based on feedback received during a previous pilot study to include more response options examining the nuanced of more frequent self-weighing [30]. Self-weighing frequency was modeled as a 6-level categorical variable, with individuals who responded, “more than once a day” collapsed into the “every day” category, due to relatively small sizes in these categories.

Mood Changes Due to Self-Weighing was measured on a 4-level Likert scale anchored by “strongly disagree” and “strongly agree” with the statement: “When I weigh myself, the number I see on the scale impacts my mood” (test-retest correlation: *r* = 0.75). For these analyses, this variable was dichotomized to respondents who “agreed” or “strongly agreed” compared to respondents who “disagreed” or “strongly disagreed”, representing those who report experiencing mood changes due to self-weighing compared to those who do not.

### Measures of other eating and weight-related characteristics

#### BMI percentile

Self-reported height (test-retest correlation: *r* = 0.98) and weight (test-retest correlation: *r* = 0.97) were used to calculate BMI. Height and weight measurements were also completed for a subsample as part of the third study wave and very high correlations with self-reported BMI were observed among both males (*r* = 0.95) and females (*r* = 0.98) [31, 32]. The following age and sex-specific BMI percentile cut-points were used: BMI < 15th percentile, 15th percentile ≤ BMI ≤ 85th percentile, BMI > 85th percentile. These cut-points were selected based on the work from Must, et al. [33].

#### Eating Disorder Diagnosis

Eating disorder diagnosis was measured with the question: “Do you currently or have you ever had an eating disorder?” (test-retest percent concordant: 98%). This item had three response options: “Yes, currently”, “Yes, recovered”, and “No, never”, and was modeled as a categorical exposure in regression models.

#### Weight Management Goal

Weight Management Goal was measured from participants’ response to the prompt, “Are you currently trying to: 1) lose weight, 2) stay the same weight, 3) gain weight, or 4) I am not trying to do anything about my weight” (test-retest percent concordant: 82%). Weight management goal was modeled as a categorical exposure in regression models, with “not trying to do anything about my weight” used as the reference category.

#### Fear of weight gain

Respondents who indicated they “strongly agree” with the statement: “I am worried about gaining weight” (test-retest correlation: *r* = 0.62) were categorized as having a fear of weight gain and all others were categorized as not being worried about gaining weight.

#### Weight/shape overvaluation

Respondents were categorized as having weight/shape overvaluation if they responded that either weight and shape were: “among the main things that affected how I felt about myself” or “were the most important things that affected how I felt about myself” to the prompt, “During the past six months, how important has your weight or shape been in how you feel about yourself?” (test-retest correlation: *r* = 0.71), or if the respondent was classified as low-weight or normal-weight but reported that they feel that they are: “somewhat overweight” or “very overweight” (test-retest correlation: *r* = 0.67). Questions were modeled after previously validated measures, and previous work in this population showing weight/shape concern to be important in the development of disordered eating behaviors [34, 35].

#### Binge eating

Respondents were categorized as engaging in binge eating if they responded “yes” to both of the following questions: “In the past year, have you ever eaten so much food in a short period of time that you would be embarrassed if others saw you? (binge eating)” (test-retest percent concordant: 90%) and “During the times when you ate this way, did you feel you couldn’t stop eating or control what or how much you were eating?” (test-retest percent concordant: 78%) [34].

#### Compensatory Behavior

Respondents who indicated that they “used laxatives” (test-retest percent concordant: 97%) or “made myself vomit” (test-retest percent concordant: 98%) to the prompt, “Have you done any of the following things in order to lose weight or keep from gaining weigh during the past year?” were classified as having engaged in compensatory behaviors [25, 36].

#### Sociodemographic characteristics

All covariates were assessed using self-reported measures. One question asked, “Are you…?” With responses of “Male” or “Female”. Age was calculated by subtracting the participants’ birthdays from date of survey completion. Participants were asked to identify their ethnic/racial background as any combination of: “White”, “Black or African American”, “Hispanic or Latino”, “Asian American”, “Native Hawaiian or Pacific Islander”, and/or “American Indian or Native American”, and categorized as follows: “White”, “African American”, “Hispanic”, “Asian”, and “Mixed or Other”. Education level ranged from “Middle school or junior high” to “Graduate or professional degree.” Total past-year pre-tax income was reported on a scale from “Less than $20,000” to “$100,000 or more”. It is important to assess both ethnicity/race and socio-economic status (as measured by education level and income) given the possibility that individuals from diverse ethnic/racial and low socio-economic backgrounds may be more likely to experience adverse health consequences due in part to racism and social inequities.

### Statistical analyses

Based on expected differences in self-weighing related behavior and mood, it was determined a priori that all statistical analyses would be run separately for males and females [9]. Demographic characteristics and frequencies of each self-weighing variable and of the other eating and weight-related variables were compared between sexes using chi-square and t-tests. Associations between self-weighing frequency and the other eating and weight-related variables were modeled using linear regression. Self-weighing frequency was modeled as a square-root transformed continuous variable because this variable was heavily right skewed. Least square means and their 95% confidence intervals for self-weighing frequency by levels of the predictor variable were calculated from the models on the square-root scale and squared to convert them back to the weekly frequency scale.

Logistic regressions were used to test whether mood change due to self-weighing was associated with self-weighing frequency or the other eating and weight-related variables. Because a large proportion of participants reported changes in mood due to self-weighing (41%), the regression coefficients from the logistic model were used to generate estimated probabilities of reporting mood changes due to self-weighing [37, 38]. The linear and logistic regression models were adjusted for age, ethnic/racial categories, educational attainment, income, and BMI percentile. Statistical analyses were conducted using SAS version 9.4.

## Results

### Sample characteristics and prevalence of self-weighing and other eating and weight-related characteristics

Among this population-based sample of young adults (Table 1), self-weighing and changes in mood due to self-weighing were highly prevalent (Table 2). Over one third of female young adults (34.5%) and one quarter of male young adults (26.8%) reported weighing themselves at least once per week, 22.5% of female young adults and 17.0% of male young adults weighing themselves more than once per week, and 7.9% of female young adults and 7.0% of male young adults weigh once per day or more. Moreover, over half of female young adults (53.0%) and one quarter of male young adults (26.5%) reported mood changes due to self-weighing. Female young adults were significantly more likely than male young adults to report self-weighing once a month or more, mood changes due to self-weighing, current or past eating disorder diagnosis, trying to manage their weight, fear weight gain, weight/shape overvaluation, and use of binge eating and compensatory behaviors.

**Table 1:** Sociodemographic characteristics of sample statistics (*n* = 1719)

	n, (%)^a^ or mean, (SD)
*Age*	
*Years: Mean [SD]*	31.0 (1.6)
*Sex*	
Male	774 (45.0%)
Female	945 (55.0%)
*Ethnicity/Race*	
White	1166 (68.3%)
African American	145 (8.5%)
Hispanic	61 (3.6%)
Asian	254 (14.9%)
Mixed or Other	81 (4.7%)
*Educational Attainment*	
High School or Less	397 (23.2%)
Two Year Degree	414 (24.3%)
Bachelor’s Degree	613 (35.9%)
Graduate or Professional Degree	286 (16.7%)
*Income*	
Less than $20,000	125 (7.4%)
$20,000–$34,999	216 (12.8%)
$35,000–$49,999	268 (15.8%)
$50,000–$74,999	383 (22.6%)
$75,000–$99,999	300 (17.7%)
$100,000 or more	402 (23.7%)

^a^Percents may not add to 100 due to rounding

**Table 2:** Prevalence of self-weighing and other eating and weight-related characteristics

	Females, *n* = 945: (n, [%]^a^ or mean, [SD])	Males, *n* = 774: (n, [%]^a^ or mean, [SD])
***SELF-WEIGHING***		
*Self-Weighing Frequency*^b^		
Once a day or more	75 [7.9%]	54 [7.0%]
A few times per week	138 [14.6%]	77 [10.0%]
Every week	113 [12.0%]	76 [9.8%]
A few times per month	132 [14.0%]	112 [14.5%]
Every month	108 [11.4%]	88 [11.4%]
Less than once a month	379 [40.1%]	367 [47.4%]
*Self-Weighing Impacts Mood*^b^		
Yes	501 [53.0%]	205 [26.5%]
No	444 [47.0%]	569 [73.5%]
***EATING AND WEIGHT-RELATED CHARACTERISTICS***		
*BMI Percentile*		
< 15th Percentile	51 [5.4%]	40 [5.2%]
15th ≤ Percentile <85th	571 [60.4%]	484 [62.5%]
≥ 85th Percentile	323 [34.2%]	250 [32.3%]
*Eating Disorder Diagnosis*^b^		
Yes, Currently	17 [1.8%]	4 [0.5%]
Yes, Recovered	85 [9.0%]	7 [0.9%]
Never	841 [89.2%]	760 [98.6%]
*Weight Management Goal*^b^		
Lose Weight	613 [64.9%]	342 [44.3%]
Gain Weight	20 [2.1%]	38 [4.9%]
Stay Same Weight	160 [16.9%]	181 [23.5%]
Nothing about Weight	152 [16.1%]	211 [27.3%]
*Fear of Weight Gain*^b^		
Yes	373 [39.6%]	131 [17.0%]
No	570 [60.5%]	641 [83.0%]
*Weight/Shape Overvaluation*^b^		
Yes	580 [61.4%]	311 [40.2%]
No	365 [38.6%]	463 [59.8%]
*Binge Eating*^b^		
Yes	155 [16.4%]	66 [8.6%]
No	788 [83.6%]	705 [91.4%]
*Compensatory Behavior*^b^		
Yes	60 [6.4%]	11 [1.4%]
No	880 [93.6%]	762 [98.6%]

^a^Percents may not add to 100 due to rounding

^b^Males differ from females significantly at p_chisquare_ < 0.05

### Correlations between eating and weight-related characteristics and frequency of self-weighing

Both female and male young adults who reported currently having an eating disorder weighed themselves more frequently than others, in analyses adjusted for sociodemographic characteristics and BMI (Table 3). Female young adults with a current eating disorder had a predicted average of weighing themselves 1.75 times per week (95% confidence interval (CI): 1.00, 2.69) as compared to 0.64 times per week (95% CI: 0.56, 0.71) for female young adults who never had an eating disorder, after adjusting for covariates. Female young adults with a BMI at or above the 85th percentile had a significantly higher predicted self-weighing frequency (0.79 times per week, 95% CI: 0.66, 0.93) compared to female young adults whose BMI was below the 15th percentile (0.35 times per week, 95% CI: 0.16, 0.60), though no other pairwise comparisons were statistically significant. We found no associations between BMI percentile category and predicted self-weighing frequency among male young adults. Among female young adults, predicted average weekly weighing frequency was higher among those who were trying to lose weight (0.85 times per week, 95% CI: 0.76, 0.96) and those trying to stay the same weight (0.56 times per week, 95% CI: 0.41, 0.73) compared to those not trying to do anything about their weight (0.23 times per week, 95% CI: 0.14, 0.35). Among male young adults, predicted average weekly weighing frequency was higher among those trying to lose weight (0.93 times per week, 95% CI: 0.80, 1.07) or gain weight (0.72 times per week, 95% CI: 0.46, 1.04) compared to those not trying to do anything about their weight (0.26 times per week, 95 CI: 0.18, 0.35). Both male and female young adults who reported any disordered eating behaviors or related cognitions (i.e., binge eating, compensatory behavior, fear of weight gain, or weight/shape overvaluation) had higher predicted self-weighing frequency as compared to those who did not report having the respective disordered eating cognitions or behaviors. For example, male young adults who used compensatory behaviors weighed themselves an average of 1.31 times per week (95% CI: 0.74, 2.05) compared to an average of 0.56 times per week (95% CI: 0.49, 0.63) among male young adults who did not report use of compensatory behaviors.

**Table 3:** Predicted average weekly self-weighing frequency by eating and weight-related characteristics (adjusted for BMI, age, ethnicity/race, sex, education and income)^a^

	Females (Mean [95% CI])	Males (Mean [95% CI])
*Eating Disorder Diagnosis*		
Yes, Currently	**1.75 [1.00 to 2.69]**^b^	**1.81 [0.68 to 3.5]**^b, d^
Yes, Recovered	0.86 [0.61 to 1.16]	0.38 [0.01 to 1.25]^d^
Never (Ref)	0.64 [0.56 to 0.71]	0.57 [0.50 to 0.64]
*BMI Percentile*^c^		
< 15th Percentile	**0.35 [0.16 to 0.60]**^b^	0.37 [0.17 to 0.65]
15th ≤ Percentile <85th	0.63 [0.55 to 0.73]	0.58 [0.49 to 0.67]
≥ 85th Percentile (Ref)	0.79 [0.66 to 0.93]	0.59 [0.48 to 0.72]
*Weight Management Goal*		
Lose Weight	**0.85 [0.76 to 0.96]**^b^	**0.93 [0.80 to 1.07]**^b^
Gain Weight	0.35 [0.12 to 0.69]	**0.72 [0.46 to 1.04]**^b^
Stay Same Weight	**0.56 [0.41 to 0.73]**^b^	0.39 [0.28 to 0.51]
Nothing about Weight (Ref)	0.23 [0.14 to 0.35]	0.26 [0.18 to 0.35]
*Fear of Weight Gain*		
Yes	**1.04 [0.90 to 1.18]**^b^	**1.00 [0.79 to 1.24]**^b^
No (Ref)	0.47 [0.40 to 0.55]	0.50 [0.43 to 0.58]
*Weight/Shape Overvaluation*		
Yes	**0.79 [0.69 to 0.89]**^b^	**0.71 [0.60 to 0.84]**^b^
No (Ref)	0.50 [0.40 to 0.60]	0.49 [0.41 to 0.57]
*Binge Eating*		
Yes	**1.26 [1.03 to 1.51]**^b^	**1.02 [0.73 to 1.36]**^b^
No (Ref)	0.58 [0.51 to 0.65]	0.53 [0.46 to 0.61]
*Compensatory Behavior*		
Yes	**1.28 [0.95 to 1.67]**^b^	**1.31 [0.74 to 2.05]**^b^
No (Ref)	0.62 [0.55 to 0.69]	0.56 [0.49 to 0.63]

^a^Frequency of self-weighing was square root transformed for the regression model. Least square means and their 95% confidence levels were squared to back calculate to the unit of reported times self-weighing per week

^b^Different from the reference level at *p* < 0.05

^c^Adjusted for Age, Ethnicity/Race, Education and Income

^d^Estimate may not be reliable due to small numbers of males with eating disorder diagnosis

### Associations between self-weighing frequency and mood changes due to self-weighing

Among females, more frequent self-weighing was consistently associated with increased likelihood of mood changes due to self-weighing (Table 4). Notably, 69% of females who weighed themselves a few times per week reported their mood was impacted by self-weighing (95% CI: 60%, 77%), as compared to 39% for females who weighed themselves less than once a month (95% CI: 34, 45%) after adjusting for covariates. Among males, a similar pattern was seen with males weighing themselves at least daily (probability = 47%, 95% CI: 35, 59%), a few times per week (probability = 34%, 95% CI: 25, 43%), and a few times per month (probability = 36%, 95% CI: 28%, 44%) being more likely to report self-weighing impacting mood compared to those who weighed less than once a month (probability = 20%, 95% CI: 16%, 24%) after adjusting for covariates.

**Table 4:** Predicted probability of mood changes due to self-weighing within levels of frequency of self-weighing, and other eating and weight-related characteristics (adjusted for BMI, age, ethnicity/race, educational attainment, and income)

	Probability that respondent reports that self-weighing impacts their mood (Females *n* = 1039)	Probability that respondent reports that self-weighing impacts their mood (Males *n* = 782)
*Self-Weighing Frequency*		
Once a day or more	**0.68 [0.55 to 0.79]**^a^	**0.47 [0.35 to 0.59]**^a^
A few times per week	**0.69 [0.60 to 0.77]**^a^	**0.34 [0.25 to 0.43]**^a^
Every week	**0.54 [0.44 to 0.64]**^a^	**0.29 [0.21 to 0.38]**^a^
A few times per month	**0.55 [0.46 to 0.64]**^a^	**0.36 [0.28 to 0.44]**^a^
Every month	**0.53 [0.43 to 0.63]**^a^	0.20 [0.14 to 0.29]
Less than once a month (Ref)	0.39 [0.34 to 0.45]	0.20 [0.16 to 0.24]
*Weight Status*^b^		
< 15th Percentile	**0.20 [0.11 to 0.34]**^a^	**0.09 [0.04 to 0.21]**^a^
15th ≤ Percentile <85th	**0.47 [0.42 to 0.52]**^a^	**0.21 [0.18 to 0.25]**^a^
≥ 85th Percentile (Ref)	0.65 [0.59 to 0.71]	0.40 [0.35 to 0.46]
*Eating Disorder Diagnosis*		
Current Eating Disorder	0.59 [0.33 to 0.80]	0.55 [0.20 to 0.86]^c^
Recovered Eating Disorder	**0.68 [0.57 to 0.78]**^a^	0.51 [0.22 to 0.80]^c^
Never had an Eating Disorder (Ref)	0.50 [0.46 to 0.54]	0.26 [0.23 to 0.29]
*Weight Management Goal*		
Lose Weight	**0.61 [0.57 to 0.66]**^a^	**0.41 [0.36 to 0.47]**^a^
Gain Weight	0.41 [0.23 to 0.62]	0.13 [0.07 to 0.24]
Stay Same Weight	0.39 [0.30 to 0.48]	**0.22 [0.17 to 0.28]**^a^
Nothing about Weight (Ref)	0.28 [0.20 to 0.37]	0.09 [0.06 to 0.13]
*Fear of Weight Gain*		
Yes	**0.77 [0.71 to 0.81]**^a^	**0.62 [0.54 to 0.70]**^a^
No (Ref)	0.36 [0.32 to 0.41]	0.20 [0.17 to 0.23]
*Weight/Shape Overvaluation*		
Yes	**0.64 [0.59 to 0.68]**^a^	**0.42 [0.37 to 0.47]**^a^
No (Ref)	0.32 [0.27 to 0.38]	0.17 [0.14 to 0.20]
*Binge Eating*		
Yes	**0.78 [0.70 to 0.85]**^a^	**0.51 [0.39 to 0.62]**^a^
No (Ref)	0.47 [0.43 to 0.51]	0.24 [0.21 to 0.27]
*Compensatory Behavior*		
Yes	**0.70 [0.56 to 0.80]**^a^	**0.90 [0.67 to 0.98]**^a^
No (Ref)	0.50 [0.47 to 0.54]	0.26 [0.23 to 0.29]

^a^Different from the reference level at p < 0.05

^b^Adjusted for Age, Ethnicity/Race, Education and Income

^c^Estimate may not be reliable due to small numbers of males with eating disorder diagnosis

### Associations between other eating and weight-related characteristics and mood changes due to self-weighing

Males and females with a BMI at or above the 85th percentile were significantly more likely to have mood changes due to self-weighing compared to those with a BMI below the 85th percentile. Females in recovery from an eating disorder were also more likely to have mood changes due to self-weighing (Probability = 68%, 95% CI: 57%, 78%) compared to those with a current eating disorder (Probability = 59%, 95% CI: 33%, 80%) or who have never had an eating disorder (Probability = 50%, 95% CI: 46%, 54%). Among males, there were no associations between eating disorder diagnosis and mood changes due to self-weighing. Both males and females who were trying to lose weight, feared weight gain, had weight/shape overvaluation, or had binge eaten or used a compensatory behavior, were more likely to indicate mood changes due to self-weighing as compared to those who did not endorse the respective eating disorder behavior or cognition.

## Discussion

This study examined the correlates of self-weighing frequency, and whether self-weighing frequency or other eating and weight-related measures (such as eating disorder diagnosis, BMI, weight management goals, and disordered eating behaviors and cognitions) are associated with mood changes due to self-weighing in a population-based sample of young adults. Self-weighing and reporting mood changes due to self-weighing were common among males and females, with over half of females and one quarter of males reported that their mood changed due to self-weighing. Male and female young adults with a current eating disorder diagnosis, those trying to lose weight, and those who reported disordered eating behaviors or cognitions weighed themselves more frequently than their counterparts, as did female young adults with higher BMI values. More frequent self-weighing was associated with increased likelihood of self-weighing affecting mood, particularly for females. Changes in mood due to self-weighing were more likely among those with a BMI above the 85th percentile, those trying to lose weight, or among those who reported any disordered eating behaviors or cognitions. Overall, these findings show that self-weighing is common, and that self-weighing affects mood for a large proportion of young adults, particularly those who are more likely to be weight-concerned.

Similar to prior work, we found self-weighing to be more frequent among those with eating disorders [39] and those with higher BMI [9, 40]. It is important to consider that there is likely overlap between these two groups, given that individuals with a higher BMI are more likely to have an eating disorder compared to individuals of lower BMI in population-based samples [41, 42]. Prior research has shown that mood changes due to self-weighing were common among a sample of predominantly Caucasian college females [24]. The present findings expand upon this research using a more socioeconomically and racially/ethnically diverse and older sample of young adults, corroborating a remarkably similar prevalence of mood changes due to self-weighing among females (53% in both population-based studies) [24]. Our results also demonstrate that a substantial proportion of males experience mood changes due to self-weighing, though a smaller proportion than females. The considerable prevalence of mood changes due to self-weighing among both males and females may be due in part to the prominence of appearance-based ideals for both males and females. However, self-weighing may be more likely to have an emotional impact on females because the ideal appearance for females is weight-centric, focusing on thinness, whereas the male appearance-ideal incorporates an increased desire for both muscularity and leanness, making weight less of a salient marker for body ideals among men [23, 43, 44]. Therefore, the number on the scale when self-weighing may be more indicative to appearance expectations for females rather than males, thereby causing weight to have more emotional valence for females.

Our results provide evidence that self-weighing may be most likely to cause mood changes for those with weight concerns. Females in recovery from an eating disorder were more likely to have mood changes due to self-weighing compared to those with no eating disorder history. Because both negative and positive emotions are a precursor for disordered eating [16, 45], our findings suggest that self-weighing may be particularly harmful for females working on recovery. Additionally, males and females with a higher BMI or who were trying to lose weight were more likely to report mood changes due to self-weighing. Current recommendations for weight loss among those with higher BMIs often include self-weighing [1]. While prior results suggest that mood may positively change if weight is lost [46], studies have found that self-weighing in the general public may actually contribute to weight gain [4, 5], thereby causing a negative change in mood. However, any direction of mood change may be construed as problematic, as the ability for the number on the scale to affect mood, irrespective of the direction, implies that there is a lack of objectivity to weight and that a sense of worth or identity is tied to weight for individuals whose mood is impacted. If the individual’s weight or direction of weight change is not in line with one’s ideals or goals, there may be a deterioration in mood and alternatively, an alignment between weight change and goals may lead to an improvement in mood. Because weight is highly variable short-term, body ideals are often unattainable and socially constructed, and long-term weight management is often unsuccessful, it stands to reason that having any emotional attachment to weight could be problematic long-term [47–49]. While counterintuitive, short-term positive changes in mood brought on by changes in weight could also be problematic. For example, in those with eating disorders who engaged in disordered eating and subsequently lost weight, their weight loss may lead to a positive change in mood, encouraging them to continue using disordered eating behaviors to pursue further weight loss and positive mood, thereby enforcing disordered eating patterns. Therefore, our results suggest that self-weighing may cause changes in mood in the populations for those who have weight concerns and those for whom self-weighing is often recommended: individuals with higher BMIs, and those trying to lose weight. Examining the types of mood changes in response to the scale in conjunction with expectancies and goals for weight, is an exciting direction for future research, as is examining the downstream consequences of mood changes due to self-weighing.

The community sample of young adults adds to the limited research on the potential consequences of self-weighing and for whom specifically frequent self-weighing may be problematic by causing changes in mood. Additionally, the large sample size allowed us to stratify analyses by sex, adding to the scant literature around self-weighing in males. However, this research is not without limitations, including the cross-sectional nature of the analysis, which does not allow for the determination of temporality of time-varying characteristics. Although Project EAT is a longitudinal study, mood changes due to self-weighing were only assessed in EAT-IV and thus we could not assess temporality of mood changes relative to the measures of eating and weight-related characteristics. Cognitive Behavioral theory has identified the reciprocal relationship between thoughts, mood and behaviors (e.g. eating behaviors, self-weighing behaviors) [20]. Due to research design, this study is not able to test the sequence or reciprocity of events but provides an important first step in understanding the complex relationships. Future research using ecological momentary assessment and randomized designs can better address the sequence of thoughts, mood, and behaviors, such as self-weighing. Additionally, our single item measure assessing the dichotomous impact of self-weighing on mood does not allow us to distinguish whether self-weighing negatively or positively impacts mood and if directionality of mood change differs across sub-populations, which is a promising direction for future research. There also may be heterogeneity in likelihood of weighing affecting mood among those that infrequently weigh, as there is likely a proportion of individuals who do not self-weigh as a form of body avoidance [50]. Items assessing weight/shape overvaluation and fear of weight gain were assessed using single item measures which may not fully capture these constructs; further research should examine the relationships between weight and shape overvaluation, fear of weight gain, and likelihood of self-weighing impacting mood, using more comprehensive scales. Due to stratification and sample size, we were also unable to examine gender minorities, which also warrants future examination. Future research should examine temporal relationships between self-weighing, mood, and behavior in young adults using longitudinal data at different time intervals (within a day, within a week, within a month, etc.), including ecological momentary assessment.

## Conclusions

In a society where quantifying and tracking weight and weight-related behaviors is highly prevalent and likely increasing, it is important to identify potential consequences of weight-related self-monitoring [51–53]. In particular for self-weighing, individuals who are more likely to be concerned with their weight are more likely to weigh themselves frequently and are also more likely to be emotionally affected by self-weighing. While self-weighing is often promoted for weight management, and may be useful for those who can receive this information without emotional attachment, self-weighing may be harmful for a significant portion of the general young adult population and particularly those who have weight concerns, such as those with higher BMIs. Therefore, it is imperative to consider the potential negative emotional implications of self-weighing when considering future public health and clinical recommendations regarding self-weighing.

## Data Availability

Please contact the Principal Investigator, Dr. Dianne Neumark-Sztainer if interested in using Project EAT data.

## Abbreviations

BMI: Body mass index
EAT: Eating and Activity in Teens and Young Adults
